# The Crosstalk of Melatonin and Hydrogen Sulfide Determines Photosynthetic Performance by Regulation of Carbohydrate Metabolism in Wheat under Heat Stress

**DOI:** 10.3390/plants10091778

**Published:** 2021-08-26

**Authors:** Noushina Iqbal, Mehar Fatma, Harsha Gautam, Shahid Umar, Adriano Sofo, Ilaria D’ippolito, Nafees A. Khan

**Affiliations:** 1 Department of Botany, Jamia Hamdard, New Delhi 110062, India; naushina.iqbal@gmail.com (N.I.); s_umar9@hotmail.com (S.U.); 2 Plant Physiology and Biochemistry Laboratory, Department of Botany, Aligarh Muslim University, Aligarh 202002, India; meharfatma30@gmail.com (M.F.); harshagautam99@gmail.com (H.G.); 3 Department of European and Mediterranean Cultures: Architecture, Environment, Cultural Heritage (DiCEM), University of Basilicata, 75100 Matera, Italy; dippolito.ilaria@libero.it

**Keywords:** oxidative stress, starch, total soluble sugars, antioxidative enzymes, hypotaurine

## Abstract

Photosynthesis is a pivotal process that determines the synthesis of carbohydrates required for sustaining growth under normal or stress situation. Stress exposure reduces the photosynthetic potential owing to the excess synthesis of reactive oxygen species that disturb the proper functioning of photosynthetic apparatus. This decreased photosynthesis is associated with disturbances in carbohydrate metabolism resulting in reduced growth under stress. We evaluated the importance of melatonin in reducing heat stress-induced severity in wheat (*Triticum aestivum* L.) plants. The plants were subjected to 25 °C (optimum temperature) or 40 °C (heat stress) for 15 days at 6 h time duration and then developed the plants for 30 days. Heat stress led to oxidative stress with increased production of thiobarbituric acid reactive substances (TBARS) and hydrogen peroxide (H_2_O_2_) content and reduced accrual of total soluble sugars, starch and carbohydrate metabolism enzymes which were reflected in reduced photosynthesis. Application of melatonin not only reduced oxidative stress through lowering TBARS and H_2_O_2_ content, augmenting the activity of antioxidative enzymes but also increased the photosynthesis in plant and carbohydrate metabolism that was needed to provide energy and carbon skeleton to the developing plant under stress. However, the increase in these parameters with melatonin was mediated via hydrogen sulfide (H_2_S), as the inhibition of H_2_S by hypotaurine (HT; H_2_S scavenger) reversed the ameliorative effect of melatonin. This suggests a crosstalk of melatonin and H_2_S in protecting heat stress-induced photosynthetic inhibition via regulation of carbohydrate metabolism.

## 1. Introduction

Heat stress is an emerging issue due to global rise in temperature which will adversely affect plants’ growth, photosynthesis, crop production and sustainability [1,2,3]. Hassan et al. [1] reported that heat stress harshly impacted the growth and quality of plant, ecosystem as well as food security. The expansion in the world’s temperature because of anthropogenic activities is the significant worry for mankind [2], as this will lead to major losses in crop production worldwide [3]. The photosynthetic ability of plants gets disturbed by the enhanced production of reactive oxygen species (ROS) under high temperature [4]. ROS adversely affect enzyme activity and membrane fluidity, resulting in altered growth and development of plants and yield loss [1]. The plants exposed to heat stress exhibit altered carbohydrate metabolism through modulation in expression of the involved genes [5]. Transgenic plants with higher levels of sugars/sugar alcohols like mannitol, fructans, trehalose and raffinose are tolerant to different stresses [5]. Plants accelerate inherent different strategies for better stress tolerance. More recently the role of melatonin in heat stress tolerance has been explored [6,7,8,9].

Melatonin is a pleiotropic signal molecule that is known to maintain balance between the different physiological processes and protection of plants besides numerous abiotic stresses responses [10,11] both through endogenous manipulation or exogenous application [8,12,13,14]. It works as an antioxidative molecule to scavenge ROS [15] and protects photosynthesis [16]. It is referred to work by means of an antioxidant that regulates abiotic stress tolerance [14]. Melatonin changes the cell layer penetrability arbitrated through ion transporters, which regulates the stomatal opening and closing in plants. Melatonin improves photosynthetic capacity of plants and upgrades the photosystem (PS) I and PS II, incites higher nitrogen and chlorophyll content and higher substance of dissolvable proteins and Rubisco [14,17]. Melatonin improved antioxidant system for enhancing tolerance to high temperature in tomato [18,19] and in wheat [9] by increasing transcription of stress-responsive genes that helped in enduring photosynthetic machinery and increasing resistance to heat stress. In view of its diverse roles, melatonin is considered as a regulator of several processes [14,20]. Moreover, melatonin shows interaction with different phytohormones and signalling molecules [17,21]. He and He [22] have reported melatonin and nitric oxide (NO) may actuate different plant physiological responses through their association under both normal or stress conditions and hydrogen sulfide (H_2_S) communicates with ROS and NO influencing the two ROS and responsive nitrogen species (RNS) uptake [23,24]. Mukherjee and Bhatla [25] found that both H_2_S and melatonin ameliorated salt stress mediated decrease in tomato growth and exogenous melatonin modulated early H_2_S signalling.

Hydrogen sulfide is the third important gasotransmitter that regulates various physiological responses under stress and plays an important role in the damaging effect of heat stress [4]. It directs germination, development, senescence and plays protective role in plants [26]. Furthermore, it can diffuse to various cells parts and maintain equilibrium the antioxidant system pools by providing sulfur to cells or changing osmolytes levels, malonaldialdehyde substance or mechanism of H_2_S biosynthesis and enzymes of antioxidants [27]. Yang et al. [28] found that H_2_S played an important role in combating heat stress and sodium hydrosulfide (NaHS)-pretreated seedlings (a H_2_S donor) reduced oxidative stress via enhancement in the activity and gene expression level of antioxidant enzymes along with soluble sugar levels and H_2_S in wheat. In *Spinacia oleracea* seedlings, H_2_S stimulates chloroplast biogenesis for the improvement of photosynthesis and the gene expression of photosynthetic enzymes and thiol redox modification [29].

During study of the photosynthetic process under heat stress, it is equally important to gather knowledge on the metabolism of carbohydrates under stress. Starch plays an important role in the tolerance of different abiotic stress [30], and works as a source-sink relations modulator [31]. Starch degradation is activated under stress and contributes to sugar accumulation [32,33]. Sugars may likewise aggregate from photosynthesis or on account of reduced demand, under development constraint of stress [30]. In transgenic plants with suppressed sucrose synthase expression, Zrenner et al. [34] in potato tubers and Tang and Sturm [35] in carrot taproots found reduced starch accumulation supporting connection between sucrose synthase and starch synthesis. Plants remobilize starch to afford carbon and energy under abiotic stress to reduce photosynthesis, but sugar released by starch degradation derived metabolites to support plant development, in addition to functioning as an osmoprotectant to mitigate stress adversities [36]. Exogenous NO and H_2_S enhanced photosynthesis and reduced the glucose-arbitrated photosynthetic repression in wheat under heat stress [4]. The work of Dawood and El-Awadi [37] on *Viciafaba*, and Wei et al. [11] on soybean elaborated that melatonin supplementation increased carbohydrates accumulation that helped in salt tolerance. Several studies report that besides acting as an antioxidant, melatonin manages worked in-related genes, comprising the metabolism of carbohydrate/fatty and amino acids [11,38,39].

The present study was initiated to find how modulation of antioxidative enzymes and carbohydrate accumulation was related to the impact of melatonin and alleviation of heat stress and consequent upon the changes in plants growth and photosynthesis. Evolution of H_2_S was also estimated and the relationship between melatonin and H_2_S for heat tolerance in wheat was worked out. Wheat was chosen because it is one of the most important crops worldwide contributing to 20% of the total calories and proteins in the human intake [40]. It contributes 30% to the world grain production and 50% to the world grain trade [41]. Wheat plants are reported to suffer heat stress [4]. Even 1 °C rise in temperature could decrease global wheat production by 6% [42] that could lead to loss in grain yield [43,44]. There is high demand for wheat, but the crop is susceptible to climate change [45]. Therefore, it is required to work out its tolerance strategies under heat stress.

## 2. Materials and Methods

### 2.1. Plant Material and Growth Conditions

The present investigation was executed on wheat (*Triticum aestivum* L. cultivar WH 542) obtained from National Seeds Corporation, New Delhi, India. The method followed by Iqbal et al. [4] was adopted to carry out the experiment.

The details of the process adopted for experimentation and procedures for determinations are given in Supplementary File S1.

### 2.2. Treatments

Plants were subjected to heat stress by exposing them for 6 h to 40 °C temperature daily during afternoon. The heat treatment was given after seedling emergence with 2–3 leaves and at 10 days after seed sowing. Heat treatment was maintained for 15 days at the same time and for the same duration. After the heat stress treatment, plants were allowed to grow at 25 °C (optimum temperature) for 5 days. The sampling time was 30 days. In the morning, the plants were supplemented with Hoagland (nutrient solution) on alternate days. Throughout the experimental period the untreated control plants were provided an optimum temperature of 25 °C. A hand sprayer was used to supply 100 µM melatonin on foliage to both heat treated or control plants at 5 days after heat stress or 15 days after seeding. A Tween-20 (0.01%) was added with both melatonin and control. To substantiate the role of H_2_S in melatonin-mediated heat tolerance, we applied 100 µM hypotaurine as H_2_S scavenger in all treatments and for all parameters studied. It was also applied at 15 days after the sowing. All concentrations taken are backed by the research of Kaya et al. [46,47]. Four replications were taken for each treatment.

### 2.3. Measurement of Growth and Photosynthetic Parameters

To measure dry weight of plants, the plants were pulled out with the root, washed to remove soil and dried on blotting paper. These plants were kept in oven at 80 °C till a constant weight was obtained and weighed for dry mass determination. Leaf area was obtained through the use of leaf area meter (LA211 Systronics, New Delhi, India).

Gas exchange photosynthetic parameters (stomatal conductance, net photosynthetic rate, and intercellular CO_2_ concentration) were measured with infrared gas analyzer (CID-340, Photosynthesis System, Bio Science, Camas, WA, USA) in the full expanded upper leaves. During the measurement photosynthetically active radiation (PAR) was 780 µmol m^−2^ s^−1^ and atmospheric CO_2_ concentrations was 390 ± 5 µmol mol^−1^ (i.e., at light saturating intensity).

SPAD chlorophyll meter (502 DL PLUS Spectrum Technologies, Plainfield, IL, USA) was used to determine chlorophyll content in the whole leaves. For chlorophyll fluorescence measurement, we used Junior-PAM chlorophyll fluorometer (Heinz Walz, GmbH, Effeltrich, Germany).

### 2.4. Determination of H_2_S, H_2_O_2_ and TBARS Content

The process demonstrated by Xie et al. [48] was used for measuring leaf H_2_S content. The generation of methylene blue from dimethyl-phenylenediamine in HCl was used for estimation of H_2_S content with slight modification.

The procedure adapted by Okuda et al. [49] was followed for the leaf H_2_O_2_ content determination. Peroxidase was added to initiate the reaction and the absorbance increase was noted at 590 nm.

Lipid peroxidation was done by estimating thiobarbituric acid reactive substance (TBARS) content through the method of Dhindsa et al. [50] and reported in Iqbal et al. [4].

### 2.5. Assay of Antioxidant Enzymes

The superoxide dismutase (SOD) activity was measured adopting Giannopolitis and Ries [51] and Beyer and Fridovich [52] method. To assay catalase (CAT), ascorbate peroxidase (APX) and glutathione reductase (GR) the method used was Aebi [53], Foyer and Halliwell [54] and Nakano and Asada [55], respectively, with little modifications. The complete process is given earlier in the study of Fatma et al. [56].

### 2.6. Determination of Enzymes Involved in Carbohydrate Metabolism

Rubisco (EC 4.1.1.39) activity was determined using the method described by Usuda [57]. The reaction was started by addition of enzyme extract to the assay medium followed by conversion of 3-phosphoglycerate to glycerol 3-phosphate and, at this stage, NADH oxidation at 30 °C at 340 nm was monitored. Protein was estimated with bovine serum albumin as standard.

Activity of sucrose phosphate synthase enzyme (EC 2.4.1.14), sucrose synthase enzyme (EC 2.4.1.13), and soluble acid invertase was assayed adopting the method of Kalwade and Devarumath [58]. The activity of ADP-Glucose pyrophosphorylase was determined by the method of Kleczkowski et al. [59]. The pyrophosphorolytic activity of ADP-Glucose pyrophosphorylase was monitored by observing the increase in absorbance at 340 nm during the conversion of NADP to NADPH at 340 nm.

### 2.7. Determination of Starch, Sucrose and Total Soluble Carbohydrate

Starch estimation was done using the method described by Kuai et al. [60], while total soluble sugars and sucrose content were measured adopting the procedure of Xu et al. [61].

### 2.8. Statistical Analysis

Analysis of data was done statistically through analysis of variance (ANOVA) by SPSS 17.0 suggested for Windows. The data are represented as treatment mean with (±) SE (*n* = 4). Least significant difference (LSD) was calculated for the significant data at *p* < 0.05. Bars showing the same letter are not significantly different by LSD test at *p* < 0.05.

## 3. Results

### 3.1. Melatonin Improves Growth and Photosynthesis under Heat Stress via H_2_S

**Figure 1:**
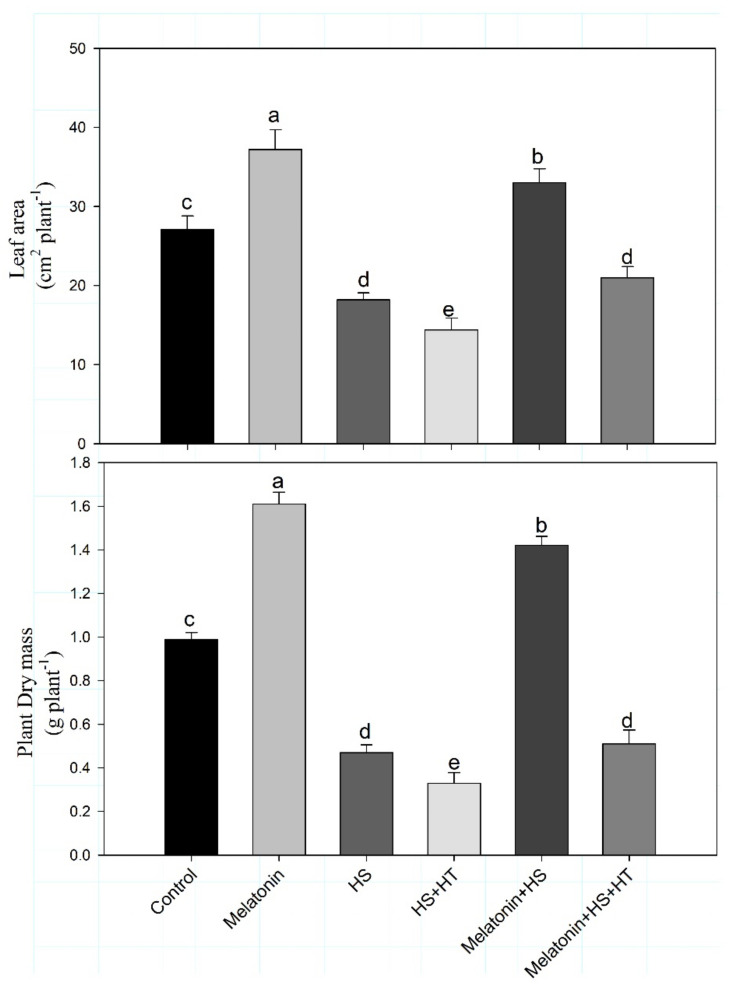
Leaf area and plant dry mass of wheat (*Triticum aestivum* L. var. WH 542) at 30 days after sowing (DAS). Plants were treated with melatonin (100 µM) and/or HT (100 µM) in the presence (40 °C) or absence (25 °C) of heat stress. Data are presented as means ± SE (*n* = 4). Data followed by the same letter are not significantly different by LSD test at *p* < 0.05. HS, heat stress; HT; hypotaurine.

We examined growth and photosynthesis parameters in wheat leaves treated with melatonin alone or in combination with heat stress in order to evaluate melatonin response under stress conditions. Substantial reductions were observed in leaf area and plant dry mass of plants by 32.8% and 52.5%, under heat stress in comparison to control. The application of melatonin individually increased plant dry mass by 62.6% and leaf area by 37.3% compared to control. The negative effect of heat stress on plant growth was altered with individual use of melatonin in heat stressed plants. The application of melatonin under heat stress proved more effective in increasing leaf area and plant dry mass by 21.7% and 43.4%, respectively, compared to control plants (Figure 1). Supplementation of hypotaurine to melatonin and heat- stressed plants resulted in reduction in leaf area and growth significantly equal to heat stress again emphasizing that melatonin action in protecting growth under heat stress was H_2_S dependent. Hypotaurine treatment to heat-stressed plants also reduced leaf area and plant dry mass which were even below the control emphasizing the importance of H_2_S in heat tolerance.

**Figure 2:**
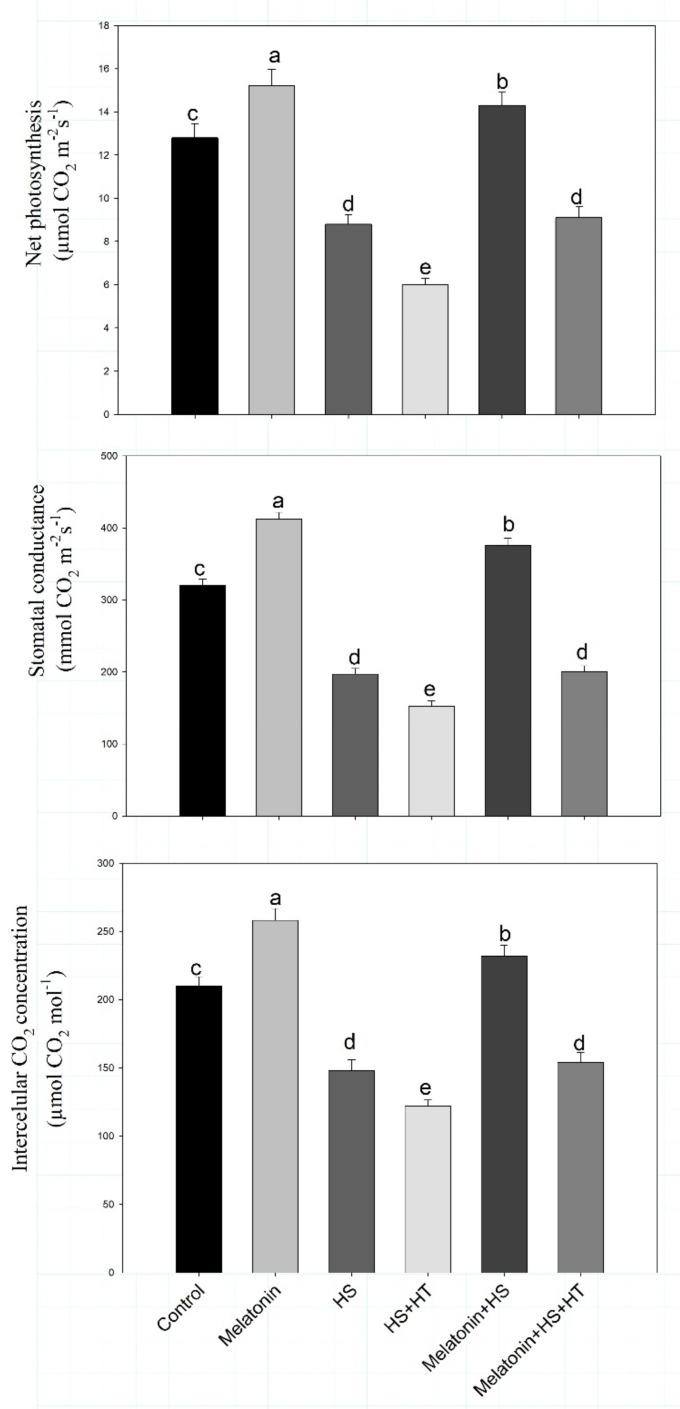
Net photosynthesis, intercellular CO_2_ concentration, stomatal conductance of wheat (*Triticum aestivum* L. var. WH 542) leaves at 30 days after sowing (DAS). Plants were treated with melatonin (100 µM) and/or HT (100 µM) in the presence (40 °C) or absence (25 °C) of heat stress. Data are presented as means ± SE (*n* = 4). Data followed by the same letter are not significantly different by LSD test at *p* < 0.05. HS, heat stress; HT; hypotaurine.

Heat stress declined net photosynthetic rate, stomatal conductance, intercellular CO_2_ concentration by 31.2%, 38.4% and 29.5%, respectively, in comparison to control. In contrast, melatonin increased net photosynthetic rate, stomatal conductance and intercellular CO_2_ concentration by 18.7%, 38.1% and 22.8% under no stress, and 11.7%, 17.5% and 10.4%, respectively, under heat stress in comparison with the untreated control plants (Figure 2).

**Figure 3:**
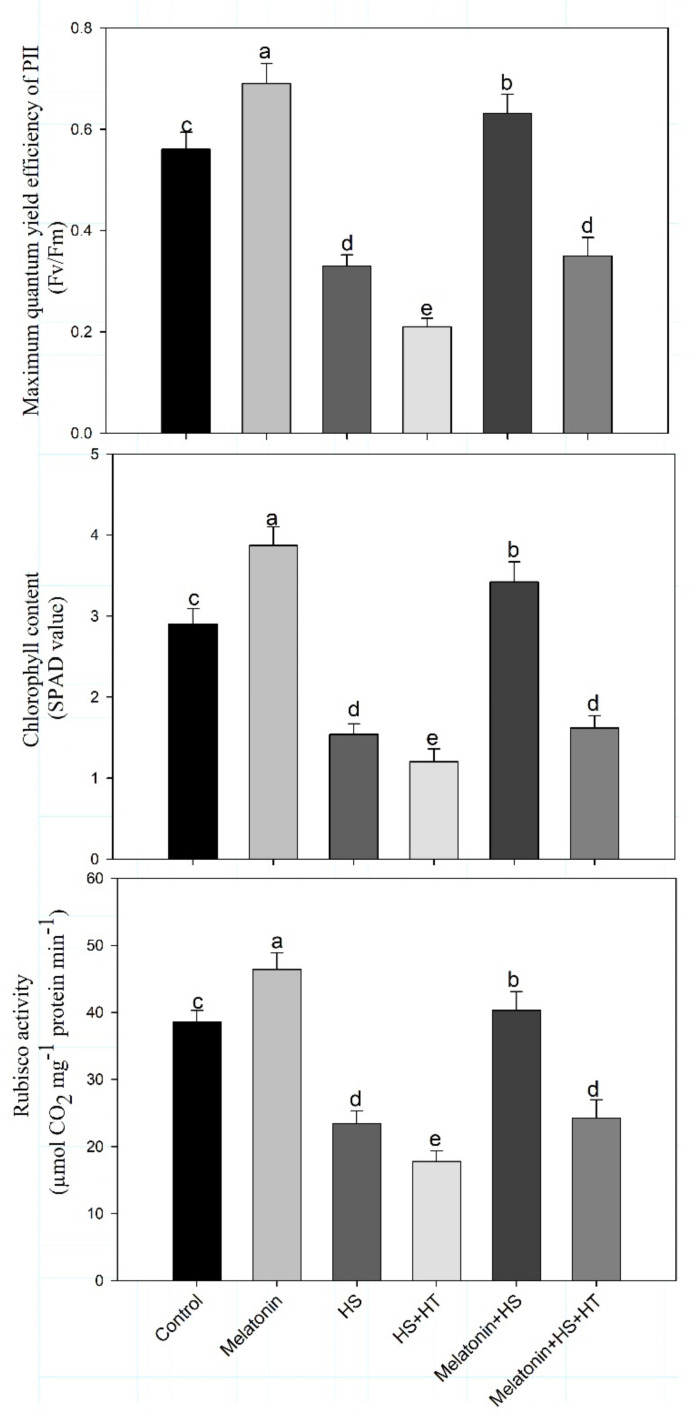
Maximum quantum yield efficiency of PS II, chlorophyll content and Rubisco activity of wheat (*Triticum aestivum* L. var. WH 542) leaves at 30 days after sowing (DAS). Plants were treated with melatonin (100 µM) and/or HT (100 µM) in the presence (40 °C) or absence (25 °C) of heat stress. Data are presented as means ± SE (*n* = 4). Data followed by the same letter are not significantly different by LSD test at *p* < 0.05. HS, heat stress; HT; hypotaurine.

Similarly, chlorophyllcontent and maximum quantum yield efficiency of PS II were also increased by 17.9% and 26.7%, respectively, with melatonin under heat stress compared to control. The maximal increase was obtained with the melatonin alone in comparison to control. The results showed that melatonin strongly reduced the negative outcome of heat stress and improved the photosynthetic parameters (Figure 3). Heat stress decreased Rubisco activity by 39.4%, while melatonin supplementation increased it by 20.2% under no stress and by 4.4% under heat stress conditions.

The importance of H_2_S in heat stress tolerance was evident from the observation that heat stress and hypotaurine treatment reduced all the photosynthetic characteristics. Photosynthetic characteristics were reduced under heat stress, but when hypotaurine was given to heat treated plants greater reduction in photosynthetic characteristics was noted. This suggests that H_2_S might act as a signalling molecule to initiate adaptation under heat stress. Hypotaurine treatment to heat-stressed plants reduced net photosynthetic rate by 31.8%, stomatal conductance by 22.8%, intercellular CO_2_ concentration by 17.6%, chlorophyll content by 17.6%, maximum quantum yield efficiency of PS II by 36.4% and Rubisco activity by 23.9% in comparison to heat stress treatment.Melatonin was thus efficient in enhancing plants photosynthetic potential under heat stress, but hypotaurine treatment reduced increases in these photosynthetic parameters even in the presence of melatonin. This suggests the role of H_2_S in melatonin mediated photosynthetic protection under heat stress.

### 3.2. Involvement of H_2_S in Melatonin-Induced Reduction of Oxidative Damage Caused by Heat Stress via Increasing Antioxidant Systems

**Figure 4:**
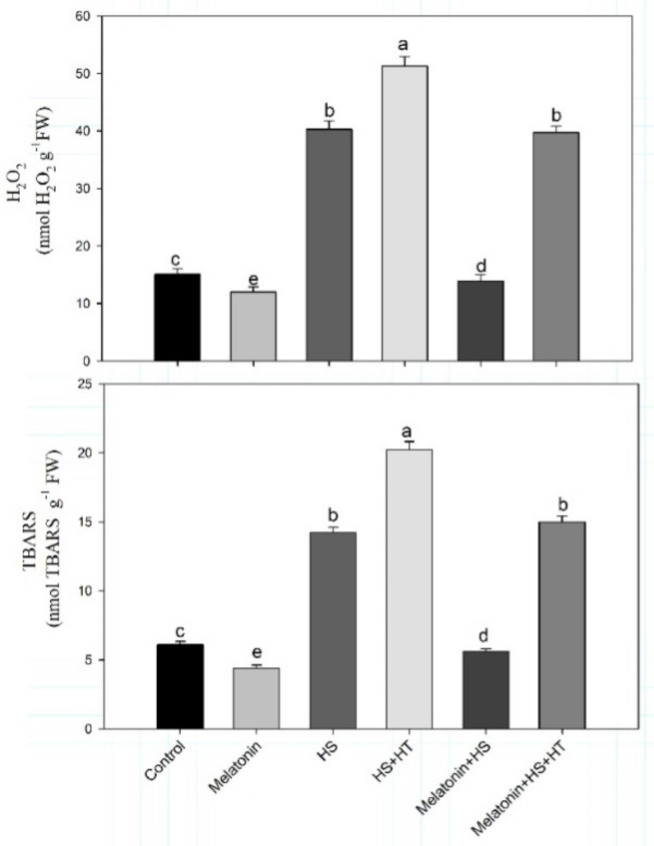
Content of H_2_O_2_ and TBARS in wheat (*Triticum aestivum* L. var. WH 542) leaves at 30 days after sowing (DAS). Plants were treated with melatonin (100 µM) and/or HT (100 µM) in the presence (40 °C) or absence (25 °C) of heat stress. Data are presented as means ± SE (*n* = 4). Data followed by the same letter are not significantly different by LSD test at *p* < 0.05. H_2_O_2_, hydrogen peroxide; HS, heat stress; HT; hypotaurine; TBARS, thiobarbituric acid reactive substances.

Exogenous melatonin was supplemented on heat-stressed wheat plants to evaluate its effect on management of oxidative stress. Heat stress led to significant increases in H_2_O_2_ and TBARS content by 2.6 and 2.3-times, respectively, over the control. Melatonin mitigated the oxidative damage caused by heat stress by decreasing H_2_O_2_ and TBARScontent by 21.8% and 16.4%, respectively, as compared to control (Figure 4), and when compared to heat stressed plants, we found that H_2_O_2_ content was reduced by 70.7% and TBARS by 64.1%. Melatonin alone maximally reduced oxidative stress with H_2_O_2_ content reduction by 33.7% and TBARS by 32.7% compared to control. Hypotaurine, H_2_S scavenger, reduced the ameliorative effect and we observed that the contents of H_2_O_2_ and TBARS were significantly equal to heat stressed plants suggesting the importance of H_2_S in melatonin-mediated reduction in oxidative stress.

**Figure 5:**
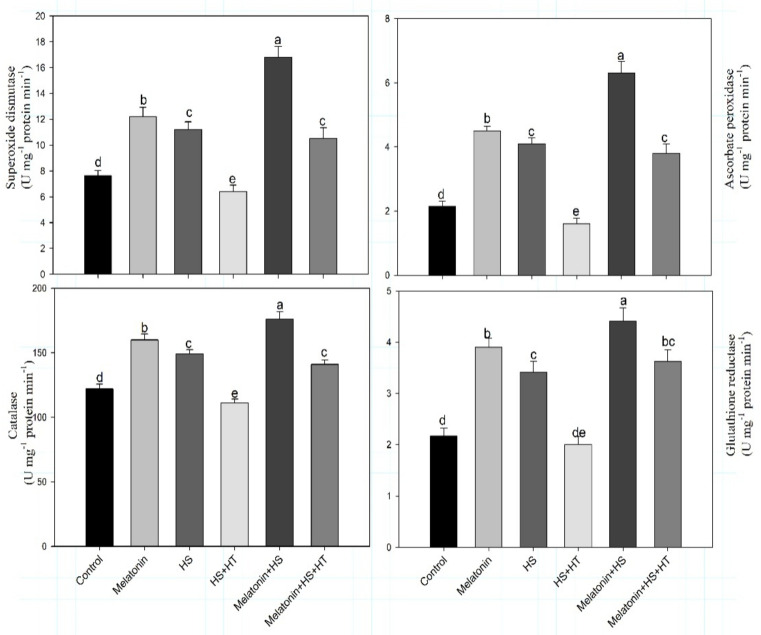
Activity of superoxide dismutase, ascorbate peroxidase, catalase and glutathione reductase of wheat (*Triticum aestivum* L. var. WH 542) leaves at 30 days after sowing (DAS). Plants were treated with melatonin (100 µM) and/or HT (100 µM) in the presence (40 °C) or absence (25 °C) of heat stress. Data are presented as means ± SE (*n* = 4). Data followed by the same letter are not significantly different by LSD test at *p* < 0.05. APX, ascorbate peroxidase; CAT, catalase; GR, glutathione reductase; HS, heat stress; HT; hypotaurine; SOD, superoxide dismutase.

The effect of melatonin was also examined on antioxidant defense system under heat stress. Heat stress increased CAT, GR, APX and SOD by 25.4%, 95.3%, 70.9% and 46.7% respectively, compared to control plants. However, exogenous application of melatonin to non-stressed plants increased the activity of CAT by 31.1%, APX by 109.3% and GR by 79.7%, and also augmented the SOD activity by 59.8% compared with control. Melatonin enhanced CAT, APX, GR and SOD activities 44.3%, 160%, 94% and 80.7%, respectively, in heat stressed plants compared to control (Figure 5). The role of H_2_S in antioxidative signalling is evident in heat stressed plants treated with hypotaurine which reduced the activity of antioxidative enzymes. It may be suggested that H_2_S induces the antioxidative defense under stress which was not sufficient to scavenge excess ROS. On reduction of H_2_S with the supplementation of hypotaurine to melatonin-treated heat stressed plants, a reduction in the antioxidative enzymes activities was observed signifying that melatonin needs H_2_S signalling for initiating antioxidative enzymes mediated defense responses.Heat stressed plants treated with hypotaurine showed the highest reduction in antioxidative enzymes activity when H_2_S content was reduced by 56.3% compared to heat stress alone.Hypotaurine caused a lesser reduction of 39.4% in the H_2_S content in the melatonin and heat stressed treated plants, however, the reduction in H_2_S resulted in reduction in the activity of antioxidative enzymes. Probably treatments resulting in higher H_2_S had higher activity of antioxidative enzymes.

### 3.3. Melatonin Enhances H_2_S under Heat Stress

**Figure 6:**
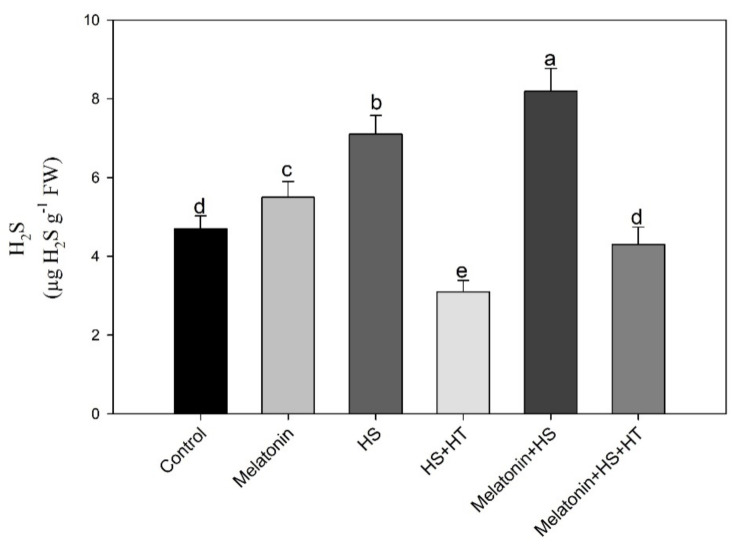
Content of H_2_S in wheat (*Triticum aestivum* L. var. WH 542) leaves at 30 days after sowing (DAS). Plants were treated with melatonin (100 µM) and/or HT (100 µM) in the presence (40 °C) or absence (25 °C) of heat stress. Data are presented as means ± SE (*n* = 4). Data followed by the same letter are not significantly different by LSD test at *p* < 0.05. HS, heat stress; HT; hypotaurine.

Heat stress enhanced H_2_S levels by 1.5-times compared with control plants. Exogenously applied melatonin further increased H_2_S in plants over control (Figure 6). However, the result was more significant with melatonin under heat stress by the evolution of leaf H_2_S by 74.5% compared to the control. These results indicate that melatonin-generated H_2_S was involved in the reduction of oxidative stress. Treatment with hypotaurine to heat stressed plants decreased H_2_S by 34.0% compared to control and 56.3% compared to heat stress. Plants supplemented with melatonin and treated with hypotaurine under heat stress showed lowering in H_2_S by 8.5% compared to control, 39.4% compared to heat stress and 47.6% compared to melatonin and heat stress treatment.

### 3.4. Involvement of H_2_S in Melatonin-Induced Alteration of Soluble Sugars and Sucrose Content

Heat stress decreased the content of total soluble sugar by 7.2% compared with the control. Conversely, melatonin treatment either alone or under heat stress increased the total soluble sugar in comparison with the control. Melatonin increased the soluble sugar content by 22.8% under non stress and by 11.2% under heat stress conditions. Thus, melatonin treatment under heat stress was beneficial to plants adaptation to environmental changes and increased the soluble sugar content. These soluble sugar contents are used to analyze the defensive ability of wheat against stress responses. In the presence of H_2_S scavenger, we observed a decrease in soluble sugars which was significantly equal to heat stress plants even in the presence of melatonin signifying that melatonin increased soluble sugars under heat stress via H_2_S.

**Figure 7:**
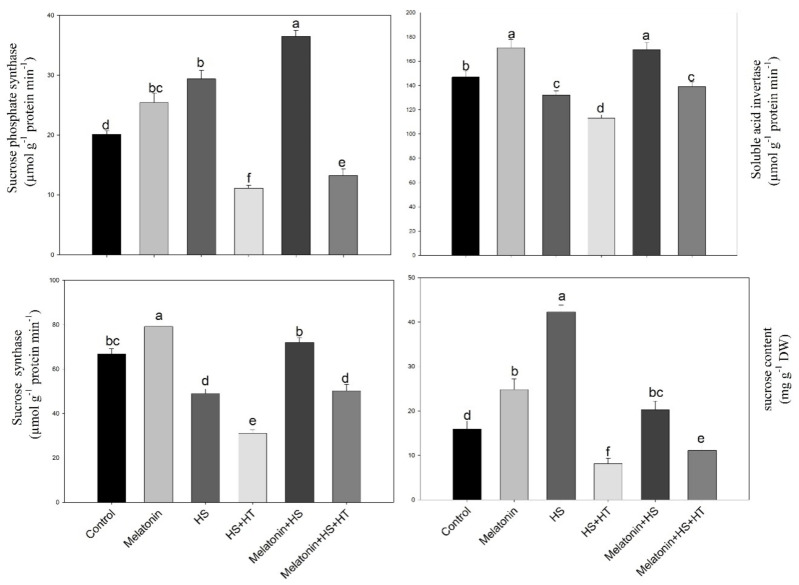
Activity of sucrose phosphate synthase, sucrose synthase, soluble acid invertase and sucrose content in wheat (*Triticum aestivum* L. var. WH 542) leaves at 30 days after sowing (DAS). Plants were treated with melatonin (100 µM) and/or hypotaurine (HT, 100 µM) in the presence of (40 °C) or absence (25 °C) of heat stress. Data are presented as means ± SE (*n* = 4). Data followed by the same letter are not significantly different by LSD test at *p* < 0.05. HS, heat stress; HT; hypotaurine.

The sucrose content was found to increase under heat stress by 2.7-times compared to the control. Exogenous melatonin enhanced sucrose content both under no stress or heat stress but in both the cases it was lower than heat-stressed plants. On hypotaurine treatment to heat treated melatonin supplemented plants, a decline in the content of sucrose below the level of control was noted and melatonin mediated increase was not observed. This suggests that melatonin requires H_2_S for its action on carbohydrate metabolism (Figure 7).

### 3.5. Requirement of H_2_S for Melatonin-Induced Effects on the Enzymes Activity in Sucrose Synthesis and Metabolism in Leaves

**Figure 8:**
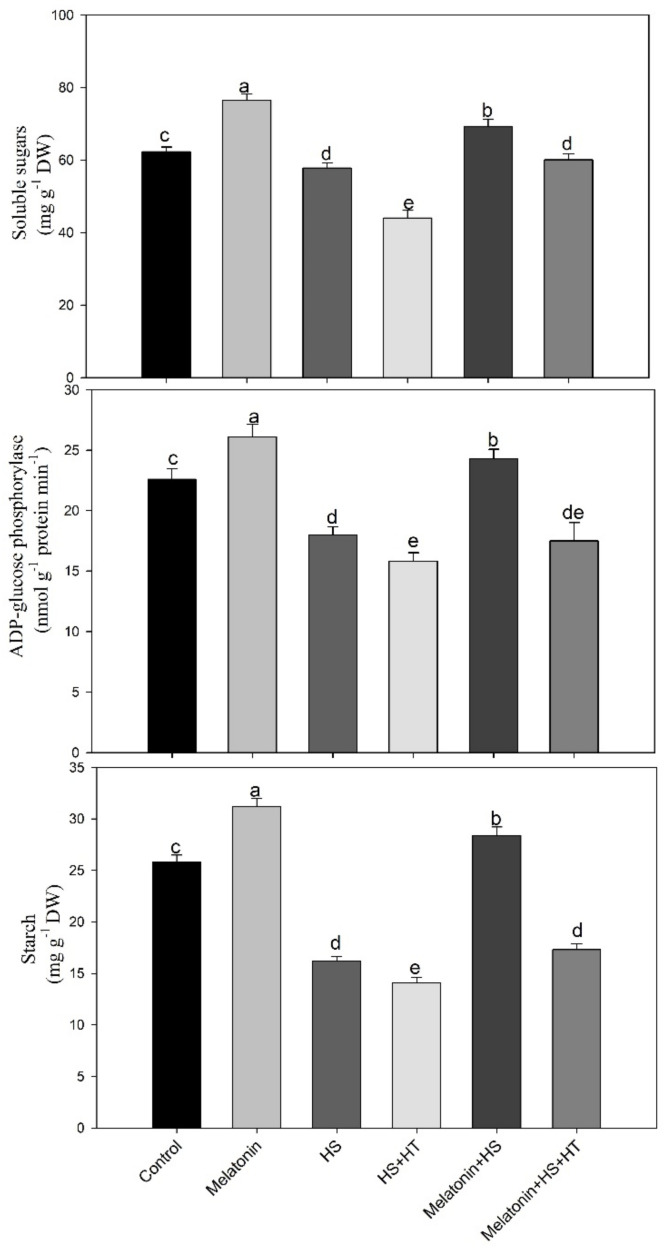
Total soluble sugars, activity of ADP-Glucose phosphorylase and starch content in wheat (*Triticum aestivum* L. var. WH 542) leaves at 30 days after sowing (DAS). Plants were treated with melatonin (100 µM) and/or hypotaurine (HT, 100 µM) in the presence of (40 °C) or absence (25 °C) of heat stress. Data are presented as means ± SE (*n* = 4). Data followed by the same letter are not significantly different by LSD test at *p* < 0.05. HS, heat stress; HT; hypotaurine.

Heat stress had significant effects on the activities of sucrose synthase, sucrose phosphate synthase, soluble acid invertase related to sucrose synthesis and cleavage (Figure 8). The activity of sucrose phosphate synthase increased by 45.8%, while adecrease in sucrose synthase by 26.7% and soluble acid invertase by 10.0% was observed in comparison to the control. The treatment of melatonin under non stress plants increased sucrose phosphate synthase by 21.2% compared to control though the increase was considerably equal to heat-stressed plants. Maximum sucrose phosphate synthase activity was noted with heat stress plants supplemented with melatonin. Activity of sucrose synthase decreased by 7.8% and acid invertase by 16.5% compared to control under heat stress, while melatonin improved it both under heat stress or no stress conditions. The content of sucrose could be increased by improving sucrose phosphate synthase activity of plants, and provide explanation of the result of increase in sugar transport.

Reduction of H_2_S by hypotaurinereduced the increase in sucrose synthesis and degradation enzymes and the decrease was comparable or lower than heat treated plants even in the presence of melatonin. Thus, H_2_S has a major role in affecting melatonin-mediated sucrose synthesis enzymes under heat stress. Reduction of H_2_S by 56.3% with hypotaurine to heat alone treated plants also reducedsucrose synthesis. The aim of using hypotaurine to heat treatment was to substantiate the role of H_2_S in heat tolerance and melatonin enhanced H_2_S evolution and mediation of tolerance, which was again reduced with hypotaurine suggesting melatonin action in heat tolerance to be mediated by H_2_S.

### 3.6. Effect of H_2_S in Melatonin-Induced Starch Accumulation and the Related Enzyme ADP-Glucose Phosphorylase

The effect of melatonin on starch accumulation and ADP-Glucose phosphorylase was examined in photosynthetic tissue of wheat plants under non stress and stress states. Heat stress decreased both accrual of starch and the activity of ADP-Glucose phosphorylase enzyme by 37.2% and 20.3%, respectively, compared to control. However, starch accumulation was increased by 18.6% in plants treated with melatonin alone and by 7.8% in plants treated with melatonin plus heat stress, as compared to control (Figure 8). Similarly, the activity of ADP-Glucose phosphorylase enzyme was increased by melatonin in non-stressed plants by 15.5%, while an increase of 7.5% was observed with melatonin under heat stressed plants in comparison to control. Application of hypotaurine reduced the melatonin-induced increase in both starch and ADP-Glucose phosphorylase activity under heat stress.Hypotaurine further decreased starch and ADP-Glucose phosphorylase activity when provided to heat alone treatment compared to heat stress.

## 4. Discussion

Plants initiate several mechanisms such as stimulation of antioxidant system, accumulation of osmolytes or secondary metabolites, heat shock proteins to resist the harmful effect of heat stress through maintenance of cellular homeostasis and repair of damaged membranes and proteins. These mechanisms are associated with production of various hormones and signalling molecules that help in adapting to heat-induced adversities. Kaya et al. [46] reported that interactive effect of H_2_S and NO resulted in reducing oxidative stress and cadmium uptake by enhancing the antioxidative enzyme system and mineral nutrients uptake. At low concentration, H_2_S affects plant development and growth by playing a major role in abiotic and biotic stress responses [27,62]. Hydrogen sulfide has gained much attention because it is a vital mode of sulfur metabolism in organisms and has increased special significance owed to its signalling properties, besides the essentiality of S requirement is known under both abiotic and biotic stress [63]. Under conditions of stress when ROS accumulation causes oxidative stress, the increased H_2_S helps in reducing the ROS via enhancing the non-enzymatic and enzymatic ways. In our study also H_2_S evolution increased under heat stress which might have signalled for increase in antioxidative mechanism because reduction of H_2_S by hypotaurin decreased antioxidative enzymes, photosynthesis, and growthunder heat stress. The rescue mechanism of H_2_S also involves signal that regulates stomatal movement, increases GSH, redox ratio, along with GSH-associated genes expression under chilling stress [64]. H_2_S relates through H_2_O_2_ and was found to upregulate ascorbate-glutathione cycle which acted as the downstream signal for H_2_S regulation on ROS [65,66]. Exogenous H_2_S application on plant seedlings was reported to inhibit oxidative stress by reducing the MDA activity, electrolyte leakage and proline [66,67] and protected photosynthesis and stablized the chloroplast structure [68]. Under heat stress accumulation of H_2_S has been reported which could be associated with the acquisition of stress tolerance in plants [69,70]. Besides, exogenously applied H_2_S can induce cross-adaptation to multiple stresses, indicating its potential as a signal molecule in cross-adaptation in plants [70,71].

The interaction of H_2_S with various other signalling molecules has also been reported. Hydrogen sulfide with Ca^2+^ and CaM effectively alleviated heat-induced damage to plants via increasing the H_2_S accumulation [72] and H_2_S-induced heat tolerance required Ca^2+^ transport toward cytoplasm then intracellular CaM coordinates it [73]. Methylglyoxal (MG) which resembles H_2_S contributes in abiotic stress response and application of MG and/or NaHS enhanced maize seedlings response to heat stress and both showed positive interaction [74]. A crosstalk of H_2_S signals with carbon monoxide, salicylic acid (SA), abscisic acid (ABA), and ethylene has also been reported [75,76,77,78], and these are found to prompt H_2_S-producing enzymes activation and endogenous H_2_S accretion under high-temperature stress. Hydrogen sulfide interacts with ethylene under osmotic stress [79], with ABA for closure of stomata [78], with NO for heat tolerance [4] with SA for lead tolerance [47], with H_2_O_2_ and brassinosteroid (BR) for stomatal closure [80] and with melatonin for salt and iron deficiency tolerance [46].

While undergoing various studies we found that melatonin interaction with H_2_S under heat stress has not been worked out, although antioxidants and photosynthesis are shown to be influenced with H_2_S application. We still do not know how H_2_S affects carbohydrate metabolism under heat stress and its relationship with melatonin. Melatonin is known as a biopromoter as it regulates numerous physiological methods and improves heat stress resistance in plants [81,82]. It is a small molecular weight indoleamine hormone explicitly being measured as a candidate phytohormone due to its various responses under biotic and abiotic stresses. Its level increases upon plant’s exposure to abiotic stresses and it scavenges ROS molecules and enhances activity of antioxidant enzyme, content of metabolite, photosynthetic efficiency and regulates stress transcription features and other metabolites and signalling molecules [83]. However, the information on how melatonin regulates carbohydrate metabolism and photosynthesis in heat stress in wheat and its interplay with H_2_S in this mechanism is scanty. In the present study, melatonin enhanced plants’ photosynthetic potential by increasing carbohydrate accumulation with enhanced antioxidative enzymes activity. The influence of melatonin was found to be dependent on H_2_S evolution as evidenced that the reduction of H_2_S reducedphotosynthesis and growth in wheat.

Similar responses in reduction of all studied photosynthetic traits were observed when H_2_S was reduced with hypotaurine under heat stress showing H_2_S signalling under heat stress as an adaptation strategy against heat adversities on plants.

### 4.1. Melatonin Increases H_2_S Evolution in Wheat under Heat Stress

Exogenous application of melatonin further increased H_2_S evolution which was induced under heat stress. Previous studies have reported that heat stress induces H_2_S evolution [4]. The interrelationship between melatonin and H_2_S has been stated in some researches. Kaya et al. [46] found that melatonin imparted resistance to join deficiency of iron and salt-stress which was intervened through both NO and H_2_S. These molecules went about as downstream signal molecule in melatonin actuated resilience. Exogenous melatonin treatment under salt stress modulated the endogenous H_2_S level and absolutely upregulated the L-cysteine desulfhydrase activity under salt stress in tomato seedlings [25]. Turk and Erdal [84] studied that melatonin application enhanced mineral element content in cold-stressed plants. Among studied nutrients, sulfur level also increased by melatonin under stress which acts as a building block of proteins, enzymes and vitamins. Sulfide is an important intermediate in sulfur metabolism and the amount of H_2_S released has been correlated with sulfate supply to the plants [71]. When hypotaurinewas supplemented to heat stressed plants with or without melatonin, reduction in H_2_S level was observed and more reduction in plants under heat stress and without melatonin. With lower level of H_2_S detected in heat stressed and hypotaurine treated plants, we observed greater reduction in all the parameters in heat stress and hypotaurine treatment.

### 4.2. Melatonin Decreases Heat Stress-Induced Oxidative Stress by Enhancing the Antioxidative Machinery: The Effect Mediated by H_2_S

In this research, heat stress was found to increase oxidative stress supported by increased TBARS and H_2_O_2_ content which was reduced by the supplementation of melatonin. Melatonin supplementation increased the activity of antioxidative enzymes (CAT, SOD, APR, GR) which scavenges the excess generated ROS to reduce oxidative stress. Enhanced activity of antioxidative enzymes detoxifies excess ROS and reduces oxidative stress. Catalase activity increased under heat stress and more increase occurred with melatonin treatment under heat stress. Catalase and peroxidises play important role in regulating the level of intracellular H_2_O_2_ and convert it into H_2_O regenerating NADP^+^ that helps plant under stress conditions [85]. Increase in CAT activity was found under heat stress in wheat; and heat tolerance in wheat genotypes was directly linked with the percent enhancement in antioxidantive enzymes including CAT, SOD and guaiacol peroxidase [86]. Reports suggest that melatonin mediates several enzymes expression that detoxify excess H_2_O_2_ such as, CAT, glutathione/ascorbate reductases, peroxidases in addition to peroxiredoxins [17,87] and balances the ROS/RNS level through antioxidant action. Either endogenous or exogenous supplied melatonin induces its own synthesis presenting melatonin as a specific regulator, an anti-stress manager and a plant master regulator [88,89]. Siddiqui et al. [90] reported that melatonin induces photosynthetic enzymes, antioxidative system, proline metabolism and carbohydrate level under salt stress. Since supplementation of melatonin increases H_2_S therefore, it was thought that melatonin action was via H_2_S. Kaya et al. [46] have studied crosstalk among H_2_S and NO in melatonin-induced salt stress tolerance in pepper. NaHS pretreatment enhances cellular viability and was found to decrease MDA accumulation and electrolyte leakage [73]. H_2_S diminishes the harmful effect of oxidative stress under heat stress [75]. In our study, we found that SOD, CAT, APX, GR activity amplified in stress and more increase was received with melatonin, but 47.6% reduction of H_2_S activity with hytotaurine in melatonin and heat stress treatment reduced the increase in antioxidative enzymes that was observed in melatonin and heat-stressed plants. Similarly, Iqbal et al. [4] reported that H_2_S enhances antioxidative enzymes activity and ascorbate-glutathione cycle to scavenge excess ROS and reduce oxidative stress in wheat under heat stress. Although not much has been discussed on the interaction between melatonin and H_2_S under heat studies, various studies support for a regulatory interaction either directly or indirectly. The relationship of melatonin, H_2_S and NO was considered in regulation of fruit ripening [91] and under salt stress by Kaya et al. [46]. However, the present reported study is the first onepertaining to melatonin and H_2_S under heat stress in wheat where melatonin increased the H_2_S generation to scavenge excess ROS by increasing the antioxidative enzymes activity.

### 4.3. Impact of Melatonin on Photosynthesis and Carbohydrate Metabolism under Heat Stress: Reversal of the Effect by H_2_S Scavenger

Heat stress affects the process of photosynthesis [4,92] by altering the activities of enzymes in metabolism of carbon and it also affects sucrose synthesis and starch accumulation by modulating explicit genes of the carbohydrate metabolism pathway [93]. The reduction in photosynthetic efficiency could be attributed to reduced activity of the Calvin cycle enzymes comprising Rubisco [94]. We found decreased Rubisco activity under heat stress which was restored by melatonin application. Reduction in chlorophyll content also leads to reduced photosynthesis probably due to increased chlorophyllase activity [95]. In the present study, the impact of melatonin was observed in reducing heat-induced photosynthetic reduction. Heat-stress reduced the content of chlorophyll, Rubisco activity in addition to efficiency of PSII and reduced stomatal conductance, intercellular CO_2_ concentration and net photosynthesis. However, melatonin supply increased Rubisco, chlorophyll content and photosynthetic traits which were reduced by application of H_2_S scavenger suggesting the involvement of H_2_S in melatonin-induced increase in photosynthesis under heat stress. Individually both melatonin and H_2_S are reported to enhance photosynthesis but here we found melatonin effect to be mediated by H_2_S. Melatonin supplementation was found to increase photosynthesis efficiency by elevating Rubisco and fructose bisphosphatase enzyme activities under heat stress in tomato seedlings [96].

We observed that heat stress led to a decrease in accumulation of starch content and total soluble sugar. Exposure of plants to heat stress can be harmful to the starch synthesis enzymes activity and starch content [97]. In several studies, ADP-Glucose pyrophosphorylase activity in maize, wheat, barley and rice were repressed under heat stress which reduced starch biosynthesis [97,98]. The present work reveals that the activity of ADP-Glucose pyrophosphorylase, one of the core rate limiting starch biosynthesis enzymes decreased in heat-stressed plants. Conversely, study also suggests that heat stress primes to rise in content of carbohydrates that helps to uphold cell turgor, helps in cell layers’ stabilization and prevents deterioration of protein [99]. Melatonin supplementation in sugar starved suspension cells of *Nicotiana tabacum* L. line Bright Yellow played regulatory role in carbohydrate metabolism byincreasing starch content through diverting the cell metabolism on gluconeogenesis whichled to carbohydrate synthesis from nonsugar precursors, like amino acids [100]. In response to stress melatonin improves plants photosynthetic ability [101,102].

Soluble sugar acts as a signalling molecule that controls growth and metabolic processes and defence linked genes to regulate plant growth [103,104]. Thus, enhanced sugar accumulation is required for potential tolerance of plants and we observed rise in sucrose under heat stress which was reduced by melatonin supplementation when the stress was relieved and photosynthesis and starch increased. The degradation of starch under heat stress was supported by reduced ADP-Glucose phosphorylase activity then increased sucrose phosphate synthase and sucrose synthase activity which helped in utilization of sugars for plant growth. Melatonin led to enhancement of starch and sucrose content to alleviate heat induced oxidative stress by maintaining an osmotic balance and providing energy and food for growth. These actions of melatonin were blocked by H_2_S scavenger which supported the role of H_2_S in increasing carbohydrate accumulation under heat stress.

**Figure 9:**
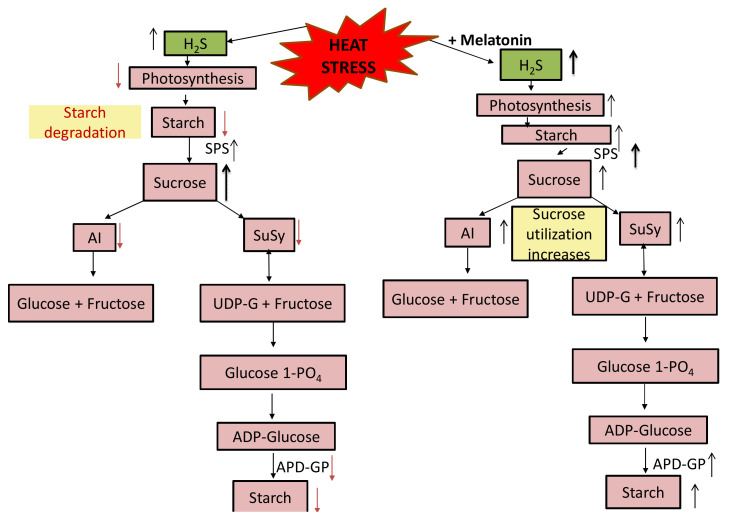
Influence of heat stress and melatonin on carbohydrate metabolism. Red arrow shows decrease and black arrow shows increase. Double headed arrow shows regulation in both the direction. Black thick arrow shows more increase. SPS, sucrose phosphate synthase; ADP-GP, ADP-Glucose phosphorylase; AI, acid invertase; SuSy, sucrose synthase; UDP-G, uridine diphosphate glucose.

Heat tolerance requires the accumulation of sugars in plants and availability of carbohydrate (glucose and sucrose), is a significant physiological attribute for the resistance to heat [105]. They reported decreased carbohydrate content under heat stress that was more in heat-susceptible genotype compared to tolerant one. In our study, the content of total soluble sugar decreased in heat stress, but by supplementation of melatonin under heat stress sugar accumulation increased. Figure 9 highlights the important changes that occur in the carbohydrate metabolism under heat stress. It may be said that plants under heat stress increase their sucrose content by favouring the degradation of starch. The conversion of sucrose to starch is reduced with reduced activity of ADP-Glucose phosphorylase but increased sucrose phosphate activity increases sucrose content. We observed that the activity of invertase and sucrose synthase reduced which resulted in reduced total soluble sugars, also with reduced starch content. The interconversion of starch-sugar in leaf is necessary for the tolerance in plants to abiotic stress [106].

Enhanced sucrose phosphate synthase activity under temperature stress helps in shifting the carbon grade starch to sucrose synthesis [107]. Melatonin increased the activity of sucrose synthase and invertase under heat stress thus leading to increased mobilization of carbon source to demanding sink. Increased expression of sucrose phosphate synthase genes is reported to increase heat tolerance. Melatonin was found to effect sucrose-phosphate synthase and sucrose synthase by either protein activation or gene transcriptions and increased invertase activity by decreasing invertase inhibitor gene expression and thus affected growth [108]. Lafta and Lorenzen [109] reported that at high temperatures, improvement in the sucrose phosphate synthase genes expression reasons upsurge in the sucrose synthesis that improves tolerance of plants under slight heat stress. Alike result was seen in our study by increased sucrose and sucrose phosphate synthase activity in heat stress. Sucrose phosphate synthase activity further increased with melatonin treatment under heat stress, however, sucrose content decreased on melatonin treatment compared to heat-stressed plants although they were greater than control. The reason for this could be the utilization of sucrose with melatonin for plants growth and development with the release of stress. Khan et al. [106] reported increased sucrose content to be aligned with decreased starch content under salt stress in tomato seedling roots. They found the effect of K^+^ in salt stress alleviation was mediated by H_2_S through its effect on carbohydrate metabolism and antioxidative defense system. The melatonin-induced increase in carbohydrate was reduced when hypotaurine was supplemented with melatonin suggesting that this carbohydrate accumulation by melatonin require H_2_S for its action under heat stress.

The increase in invertase activity with melatonin helps in heat tolerance and this increase was mediated through H_2_S. Siddiqui et al. [90] stated that melatonin alleviated salt stress through osmoregulation via increasingthe content of total soluble carbohydrate and proline and upregulated carbohydrate metabolism. Increased invertase activity enhanced sucrose transport and was found to improve heat tolerance [73].

Hydrogen sulfide was found to increase glucose utilization under heat stress in wheat and decreased heat-induced reduction in photosynthesis [4]. NaHS treatment to *S. oleracea* leaves increased accumulation of glucose and sucrose though it was lower than control; nevertheless, trehalose and fructose contents considerably increased above the control suggesting the role of trehalose and fructose in drought tolerance. Increased photosynthesis and PSII efficiency in *S. oleracea* under drought stress on H_2_S supplementation was due to modulation of sugar metabolism [69]. Ye et al. [74] found that H_2_S increases photosynthesis by promoting Rubisco activity plus photosynthetic electron transport increases chlorophyll biosynthesis. It also modulates Fv/Fm ratio, chlorophyll content together with net photosynthetic rate, stomatal conductance, and transpiration rate [65,110]. Khan et al. [106] reported that potassium and endogenous H_2_S regulated sugar metabolism for initiating adaptive responses under salinity stress. Hydrogen sulfide was found to alleviate salt stress and increased sucrose synthase, sucrose phosphate synthase, invertase activity and decreased starch content.

Although not much study has been done on the role of H_2_S in regulating carbohydrate, here we observed that the reduction of H_2_S by 47.6% by its scavenger resulted in decreased melatonin function on carbohydrate content suggesting the notion that melatonin effect on carbohydrate is via H_2_S. Taken together, our studyshows the importance of H_2_S in melatonin-induced heat stress tolerance through enhancing carbohydrate metabolism under heat stress. It may be emphasized that melatonin modulated carbohydrate metabolism under heat stress to regulate tolerance of heat and caused reduction in oxidative stress and anincrease in antioxidative metabolism via H_2_S. Possibly, melatonin functions upstream of H_2_S in inducing heat stress signalling response. Such study of regulation of carbohydrate metabolism by H_2_S in heat stress is not reported till date and this explores the possibility of H_2_S application as heat stress mitigator in plants. In order to make the concept clear, a diagrammatic representation of the effect of melatonin and H_2_S on carbohydrate metabolism under heat stress is given (Figure 9).

## 5. Conclusions

Heat stress affects carbohydrate metabolism and reduces photosynthesis. However, we observed increase in H_2_S under heat stress which could have signalled for increase in antioxidative enzymes and it was found that 56.3% scavenging H_2_S by hypotaurine reduced antioxidative enzymes activity and further lowered photosynthesis and growth. However, there was an increase in sucrose upon heat treatment which was again a defense strategy against stress, and it was observed that hypotaurine inhibited this increase in heat stressed plants. This suggests that H_2_S plays an essential role in inducing heat tolerance. Melatonin supplementation either alone or under heat stress was found to increase H_2_S evolution substantially and promoted growth. This study emphasised that melatonin helps the plants under heat stress by not only decreasing the oxidative stress through enhancement in antioxidative enzymes activity but also increases photosynthesis and growth through anincrease in the carbohydrate utilization to support growth under adverse conditions. This effect of melatonin was found to be mediated via H_2_S and reductionof H_2_S inhibited melatonin-mediated increase in heat tolerance by inhibiting the activity of antioxidative enzymes, photosynthesis and carbohydrate metabolism in wheat suggesting of downward signalling of H_2_S in melatonin-induced heat tolerance.

## Data Availability

The data presented in this study are available in the graphs provided in the manuscript.

## Supplementary Materials

The following are available online at https://www.mdpi.com/article/10.3390/plants10091778/s1, Material and methods details: plant material and growth conditions, leaf crude extracts for enzymatic assays, Determination of H_2_S, H_2_O_2_ and TBARS content, determination of Rubisco activity, determination of Starch and total soluble sugars and sucrose content, estimation of activity of sucrose synthase, sucrose phosphate synthase, acid invertase and UDP-glucose phosphorylase.
