# Supplementary material for: The Crosstalk of Melatonin and Hydrogen Sulfide Determines Photosynthetic Performance by Regulation of Carbohydrate Metabolism in Wheat under Heat Stress
Source: Plants (Basel). 2021 Aug 26;10(9):1778. doi: 10.3390/plants10091778 (PMC8465529; doi:10.3390/plants10091778)
Supplement: Supplementary file 1 [file plants-10-01778-s001.zip › plants-1276080-supplementary file.pdf]

The Crosstalk of Melatonin and Hydrogen Sulfide Determines Photosynthetic Performance by the Regulation of Carbohydrate Metabolism in Wheat under Heat Stress

Noushina Iqbal[2], Mehar Fatma[1], Harsha Gautam[1], Shahid Umar[2], Adriano Sofo[3,*], Ilaria D'ippolito[3,]
Nafees A. Khan[1*]

1 Department of Botany, Jamia Hamdard, New Delhi-110062, India; naushina.iqbal@gmail.com (N.I.); s_umar9@hotmail.com (S.U.)
2 Department of Botany, Aligarh Muslim University, Plant Physiology and Biochemistry Laboratory, Aligarh-202002, India; meharfatma30@gmail.com (M.F.); harshagautam99@gmail.com (H.G.);naf9.amu@gmail.com (N.A.K.)
3 Department of European and Mediterranean Cultures: Architecture, Environment, Cultural Heritage (DiCEM), University of Basilicata, 75100 Matera, Italy; adriano.sofo@unibas.it (A.S); dippolito.ilaria@libero.it (I.D.)

Material and Methods Details

Plant material and growth conditions

Healthy seeds of wheat (*Triticum aestivum* L.) cultivar WH 542 were treated with 0.01% $HgCl_2$ followed by washing with double distilled water to remove any adhered chemical after sterilization. Sterilized seeds were sown in pots filled with acid-washed purified sand. All pots were placed in an environmental growth chamber (Khera KI-261) and day/night temperatures were maintained at 25/18 °C, with a 12 h photoperiod (PAR 300 µmol m-2 s-1), and at a relative humidity of 65 ± 5%. Two plants per pot were maintained and were saturated every alternate day with 300 mL of full-strength Hoagland's nutrient solution. The temperature stress treatment was given by subjecting the plants to 40 °C for 6 h daily for 15 d, and they were then allowed to recover at optimum temperature (25 °C) and grown for the experimental period. The control plants were maintained throughout the experimental growth period at 25 °C.

Leaf crude extracts for enzymatic assays

Fresh leaves (200 mg) were homogenized with an extraction buffer containing 0.05% (v/v) Triton X-100 and 1% (w/v) PVP in potassium phosphate buffer (100 mM, pH 7.0) using a chilled mortar and pestle. The homogenate was centrifuged at 15,000x g for 20 min at 4 °C. The supernatant obtained after centrifugation was used for the activity assay of the different enzymes.

Determination of H_2S, H_2O_2 and TBARS content

The content of H_2S was measured in leaves according to the formation of methylene blue from dimethyl-p-phenylenediamine in HCl as described by Xie et al. (2014), with slight modifications. Leaves (0.7 g) were ground in 2.5 mL of Tris-HCl (20 mM L^{-1}, pH 6.8) containing 10 mM L^{-1} ethylene diamine tetraacetic acid (EDTA). The homogenate obtained was centrifuged at 4°C and 12,000x g for 15 min. In the supernatant (0.75 mL), 0.2 mL of 1% (w/v) zinc acetate was added for trapping H_2S. It was allowed to develop for 30 mins and then 0.1 mL of 20 mM L^{-1} dimethyl-p-phenylenediamine dissolved in 7.2 mol L–1 HCl and 0.1 mL of 30 mM L–1 ferric chloride in 1.2 mol L^{-1} HCl were added. The formation of methylene blue was determined spectrophotometrically at 670 nm. Different concentrations of NaHS were used as the standard curve expressed as nmol g^{-1} fresh weight (FW).

Leaf H_2O_2 content was determined by following Okuda et al. (1991). Fresh leaves (500 mg) were ground in ice-cold 200 mM $HClO_4$ (perchloric acid) to obtain homogenate which was centrifuged at 1500× g for 10 min and then $HClO_4$ of the supernatant was neutralized with the addition of 4 M KOH. Subsequently, another centrifugation was performed at 500× g for 3 min to eliminate the insoluble $KClO_4$. The reaction mixture contained 1.5 mL of eluate, 400 µL of 12.5 mM 3- dimethyl aminobenzoic acid (DMAB) in 0.375 M phosphate buffer (pH 6.5), 80 µL of 3-methyl-2- benzothiazoline hydrazone

and 20 μL of peroxidase (0.25 unit). The reaction was started by the addition of peroxidase at 25 °C and the increase in absorbance was recorded at 590 nm.

The content of TBARS (thiobarbituric acid reactive substances) was estimated by the method of Dhindsa et al. (1981), which provided the status of lipid peroxidation in leaves. Fresh leaves (500 mg) were homogenized in 0.25% 2-thiobarbituric acid in 10% trichloroacetic acid and the mixture was heated at 95 °C for 30 min and then rapidly cooled in an ice bath. The mixture was centrifuged at 10,000x g for 10 min. To 1 mL aliquot of the supernatant, 4.0 mL of 20% trichloroacetic acid containing 5% thiobarbituric acid was added, and the final color intensity was recorded at 532 nm.

Determination of Rubisco activity

Rubisco activity was determined by monitoring NADH oxidation at 340 nm that occurs at 30 °C and after the addition of enzyme extract to the reaction mixture, resulting in the conversion of 3-phosphoglycerate to glycerol-3-phosphate (Usuda 1985). For the extraction of the enzyme, 1.0 g of leaf tissue were subjected to ice-cold extraction buffer that consisted of 0.25 M Tris-HCl (pH 7.8), 0.0025 mM EDTA, 0.05 mM $MgCl_2$, and 37.5 mg DTT and was homogenized in a chilled mortar and pestle. The homogenate was centrifuged at 10,000 g for 10 min at 4 °C. The supernatant obtained after centrifugation was used to assay the enzyme. The reaction mixture contained 100 mM Tris-HCl (pH8.0), 10 mM $MgCl_2$, 40 mM $NaHCO_3$, 4.0 mM ATP, 0.2 mM NADH, 5.0 mM DTT, 0.2 mM EDTA, 1.0 U of glyceraldehydes-3-phosphodehydrogenase, 1.0 U of 3-phosphoglycerate-kinase and 0.2 mM of ribulose-1,5-bisphosphate.

Determination of Starch and total soluble sugars and sucrose content

Dried leaf tissues were ground and filtered using a 1 mm sieve. The powdered material (0.1 g) was put into a 10 mL centrifuge tube and mixed with 5 mL of 80% ethanol. The mixture was incubated in a water bath shaker at 80 °C for 30 min, and then centrifuged at 4000 rpm for 5 min. The pellets were extracted using 80% ethanol. Ethanol was removed through evaporation. Starch in the residue was released in a boiling bath with 2 mL of distilled water for 15 min and then cooled to room temperature. Then, leaf starch was hydrolyzed with 9.2 mol L^{-1} $HClO_4$ (2 mL) for 15 min. Distilled water (4 mL) was added into the samples, and samples were then centrifuged at 4000 rpm for 10 min. The residue was extracted one more time using 4.6 mol L^{-1} $HClO_4$ (2 mL). The supernatants were retained, combined, and mixed with distilled water until a volume of 25 mL was reached. The starch concentration was measured spectrophotometrically at 620 nm using an anthrone reagent and glucose was used as the standard.

For the estimation of total soluble sugar, fully expanded top leaves were collected for each treatment. Leaf samples were oven-dried at 80 °C and ground into a fine powder. The dried sample (100 mg) was extracted using 10 mL of 80% ethanol and kept in a water bath at 80-85 °C for 30 min. The extract was centrifuged, and the supernatant was transferred to a 100 mL volumetric flask, and the extraction was repeated three times. Alcohol extract was evaporated in a water bath at 80-85°C. All three supernatants were pooled in the flask followed by the addition of distilled water to reach a volume of 100 mL. An aliquot of the extract was used for the determination of soluble sugars with anthrone reagent, and the absorbance of reaction mixture was monitored at 630 nm using a spectrophotometer.

For sucrose content estimation, each reaction contained 50 mM of UDP-glucose, 50 mM of extraction buffer, 10 mM of MgCl2 and 200 L of extract in a total volume of 550 L. The reaction was initiated by incubating the enzyme extract at 30 °C for 30 min. The reaction was stopped using 100 L 2

mol L^{-1} of NaOH and heating the solution for 10 min at 100 °C to destroy unreacted hexoses and hexose phosphates. The solution was then cooled and mixed with 1 mL of 0.1% (w/v) resorcin in 95% (v/v) ethanol and 3.5 mL of 30% (w/v) HCl before being incubated for 10 min at 80 °C. Sucrose content was calculated from a standard curve measured at A480 nm.

Estimation of activity of sucrose synthase, sucrose phosphate synthase, acid invertase and UDP-glucose phosphorylase

The sucrose synthase and sucrose phosphate synthase activity assays were conducted at 37 °C in the direction of sucrose synthesis at pH 7.5. Two hundred microliters of desalted enzyme extract was added to 200 µL of assay solution. The sucrose synthase activity assay solution contained 50 mM Hepes (pH 7.5), 15 mM $MgCl_2$, 25 mM Fru, and 25 mM UDP-Glc. The SPS assay solution contained 100 mM Hepes (pH 7.5), 20 mM Glc-6-P, 4 mM Fru-6-P, 3 mM UDP-Glc, 5 mM MgCl2, and 1 mM EDTA. For control, UDP-Glc was not added in the assay solutions. The reactions were maintained for an incubation time of 30 min for SS and 60 min for SPS and then immediately stopped by boiling for 3 min. Enzyme reaction volume and freshly prepared reagents were proportionally increased for the spectrophotometric readings at 630 nm. The enzyme assay was measured in micromoles per minute per gram of protein.

Acid invertase activity was assayed at 37 °C by adding 150 µL of desalted enzyme extract to an equal volume of 1 M sodium acetate (pH 4.5) and 300 µL of 120 mM sucrose solution. The concentration of liberated glucose was determined using the Somogyi method (1952). The enzyme assay was expressed in micromoles per minute per gram of protein.

For the determination of ADP-glucose phosphorylase, 0.5 g of dried leaves were homogenized in a prechilled pestle and mortar at 40 °C with 2 mL of cold buffer on ice. The extraction buffer employed was composed of 50 mM 3-N-morpholino propane sulphonic acid (MOPS) pH 7.4, 2 mM $MgCl_2$, 1 mM EDTA and 2 mM dithiothritol (DTT). The homogenate so obtained was centrifuged at 10,000 x g for 10 min in a refrigerated centrifuge at 40 °C. The supernatant was used as a grain extract for enzyme analysis. The reaction was started by the addition of 200 µl of sodium pyrophosphate (2.5 µmole). The pyrophosphorolytic activity of ADP-GP was assayed spectrophotometrically by monitoring the increase in absorbance due to the conversion of NADP to NADPH at 340 nm
